# Paternal psychological response after ultrasonographic detection of structural fetal anomalies with a comparison to maternal response: a cohort study

**DOI:** 10.1186/1471-2393-13-147

**Published:** 2013-07-12

**Authors:** Anne Kaasen, Anne Helbig, Ulrik Fredrik Malt, Tormod Naes, Hans Skari, Guttorm Nils Haugen

**Affiliations:** 1 Department of Health, Nutrition and Management, Oslo and Akershus University College of Applied Sciences, P.O.Box 4, St. Olavs Plass, NO-0130 Oslo, Norway; 2 National Resource Centre for Women’s Health, Oslo University Hospital, Oslo, Norway; 3 Department of Obstetrics, Oslo University Hospital, Oslo, Norway; 4 Department of Neuropsychiatry and Psychosomatic Medicine, Oslo University Hospital, Oslo, Norway; 5 University of Oslo, Oslo, Norway; 6 Nofima Mat, Ås, Norway and University of Copenhagen, Copenhagen, Denmark; 7 Department of Gastrointestinal- and Pediatric Surgery, Oslo University Hospital, Oslo, Norway

**Keywords:** Fetal anomaly, Paternal psychological response, Pregnancy, Prenatal diagnosis, Psychological distress, Ultrasonography

## Abstract

**Background:**

In Norway almost all pregnant women attend one routine ultrasound examination. Detection of fetal structural anomalies triggers psychological stress responses in the women affected. Despite the frequent use of ultrasound examination in pregnancy, little attention has been devoted to the psychological response of the expectant father following the detection of fetal anomalies. This is important for later fatherhood and the psychological interaction within the couple. We aimed to describe paternal psychological responses shortly after detection of structural fetal anomalies by ultrasonography, and to compare paternal and maternal responses within the same couple.

**Methods:**

A prospective observational study was performed at a tertiary referral centre for fetal medicine. Pregnant women with a structural fetal anomaly detected by ultrasound and their partners (study group,n=155) and 100 with normal ultrasound findings (comparison group) were included shortly after sonographic examination (inclusion period: May 2006-February 2009). Gestational age was >12 weeks. We used psychometric questionnaires to assess self-reported social dysfunction, health perception, and psychological distress (intrusion, avoidance, arousal, anxiety, and depression): Impact of Event Scale. General Health Questionnaire and Edinburgh Postnatal Depression Scale. Fetal anomalies were classified according to severity and diagnostic or prognostic ambiguity at the time of assessment.

**Results:**

Median (range) gestational age at inclusion in the study and comparison group was 19 (12–38) and 19 (13–22) weeks, respectively. Men and women in the study group had significantly higher levels of psychological distress than men and women in the comparison group on all psychometric endpoints. The lowest level of distress in the study group was associated with the least severe anomalies with no diagnostic or prognostic ambiguity (p < 0.033). Men had lower scores than women on all psychometric outcome variables. The correlation in distress scores between men and women was high in the fetal anomaly group (p < 0.001), but non-significant in the comparison group.

**Conclusion:**

Severity of the anomaly including ambiguity significantly influenced paternal response. Men reported lower scores on all psychometric outcomes than women.

This knowledge may facilitate support for both expectant parents to reduce strain within the family after detection of a fetal anomaly.

## Background

In Norway about 98% of pregnant women attend the routine ultrasound examination at 18 weeks of gestation. Detection of fetal structural anomalies triggers psychological stress responses in the women affected [1]. The expectant fathers will also be emotionally affected, not in the least because most men are present at these ultrasound examinations. The father’s response will most likely influence the mother’s response (and vice versa), according to observations made in studies on responses to stressful life events in couples [2]. Nevertheless, little is known about men’s psychological reaction following the detection of a fetal anomaly in their partner’s pregnancy, including the influence of background variables on these responses. In a study by Skari et al. [3], men reported lower postpartum levels of distress than women after prenatal detection of a fetal anomaly. The responses were stronger than those seen if the anomaly was detected after birth. However, that study did not address the association between the psychological response and the severity of the anomaly, and all distress assessments were performed postpartum.

From studies addressing the impact of routine ultrasound examination on the psychological well-being of expecting parents, we know that men report less distress symptoms compared to women [4]. In these studies, also non-pregnancy related variables are predictors of distress responses (e.g. poor marital relationship and poor social networks) [5], [6]. On the other hand, expectant fathers with insufficient information about pregnancy and childbirth showed somewhat increased risk of being distressed [7]. However, one study conducted among partners with a child with congenital anomaly reported no significant difference in overall burden and grief between mothers and fathers one year after birth [8].

### Objective

In a previous study we showed that maternal psychological distress shortly after detection of a fetal malformation was related to the severity of the anomaly, diagnostic and prognostic ambiguity, and gestational age [1]. The aim of the present study was to describe the acute psychological and behavioural responses, i.e. social dysfunction, health perception (somatic symptoms) and psychological distress (symptoms of intrusion, avoidance, arousal, anxiety and depression) in partners of pregnant women shortly after detection of a fetal structural anomaly, and compare these results to partners of women with normal ultrasound findings. We hypothesised that the distress level was related to severity of the anomaly, diagnostic and prognostic ambiguity, and advanced gestational age. We also expected higher levels of psychological and behavioural distress compared to those with normal ultrasound findings. Men were expected to report lower scores than their partners.

## Methods

### Participants

Pregnant women and their partners were included consecutively from May 2006 to February 2009. Convenience sampling for both groups was undertaken, i.e. inclusion of subjects was not performed during vacations and in periods with heavy clinical workload. This paper concerns the responses of the fathers. The women (and their partners) were excluded if they were not fluent in Norwegian, were less than 18 years old, had an overt psychiatric diagnosis (e.g. psychosis, severe bipolar disorder, or drug abuse), or the woman expected multiplets.

The study group consisted of 155 male partners of pregnant women with confirmed fetal structural anomalies detected by ultrasound. The women were referred to our tertiary referral centre based on suspicion of a structural fetal anomaly during obstetric ultrasound examination after 12 gestational weeks (median 134, range 84–269 gestational days).

The comparison group included 100 male partners of women scheduled for delivery at our hospital. They had no history of fetal anomalies or severe obstetric complications, and were included following a routine ultrasound scan with normal findings (median 136, range 90–155 gestational days).

Medical and obstetric history, socio-demographic variables, the time interval between the suspicion of an anomaly at the referring hospital (or the normal ultrasound scan in the comparison group) and psychometric assessment, the tentative diagnosis, and gestational age were recorded as previously described [1].

### Ultrasound examination and counseling

The ultrasound examinations at our tertiary centre were performed by consultants in fetal medicine. Before inclusion all couples were counselled by the fetal medicine specialists and specialists in pediatrics, pediatric surgery, pediatric cardiology, medical genetics, or neurosurgery, as required.

### Classification of fetal anomaly

Fetal diagnoses were classified according to severity and significant prognostic or diagnostic ambiguity at the time of inclusion. Ambiguity was defined as: a) the anomaly had significant inherent prognostic variation, or b) a definite diagnosis was dependent on the results of further investigations (e.g. an invasive test). See Table 1, footnotes. If the results of further investigations were assumed to be important for the diagnosis or prognosis, severity was categorised as “severity not classified; anomaly awaiting clarification”. Fetal diagnoses were classified as previously described in Kaasen et al. [1], see Table 1. Classification was performed by three of the authors. Inter-rater agreement was high (κ = 0.86); for details see [1], [9].

**Table 1:** **Characteristics for men and women in groups with and without fetal anomaly**, **shortly after detection of fetal anomaly or normal ultrasound scan**

	**Fetal anomaly (n = 155)**			**No fetal anomaly (n = 100)**			**With and without fetal anomaly (n = 255)**	
							**Men versus men**	**Women versus women**
	**Men**	**Women**	**P-value**	**Men**	**Women**	**P**-**value**	**P**-value*	**P**-value*
	**N (%)**	**N (%)**		**N (%)**	**N (%)**			
**Age**								
19–30 years	55 (35.5)	74 (47.7)	P < 0.001	24 (24.0)	46 (46.0)	P < 0.001	P = 0.121	P = 0.372
31–35 years	53 (34.2)	60 (38.7)		44 (44.0)	34 (34.0)			
36–67 years	46 (29.7)	21 (13.5)		32 (32.0)	20 (20.0)			
Missing data	1 (0.6)							
**Education**								
Men and women < junior college	46 (29.7)		P < 0.001	10 (10.0)		P < 0.001	P < 0.001	P = 0.003
Men < junior college, women ≥ junior college	35 (22.6)			8 (8.0)				
Men ≥ junior college, women < junior college	9 (5.8)			8 (8.0)				
Men and women ≥ junior college	62 (40.0)			72 (72.0)				
Missing data	3 (1.9)			2 (2.0)				
**Previous children**								
Men and women no previous children	63 (40.6)		P < 0.001	55 (55.0)		P < 0.001	P = 0.213	P = 0.067
Men previous children, women no previous children	9 (5.8)			3 (3.0)				
Men no previous children, women previous children	10 (6.5)			1 (1.0)				
Men and women previous children	71 (45.8)			40 (40.0)				
Missing data	2 (1.3)			1 (1.0)				
**Married or cohabiting**	150 (96.8)		n. a.	100 (100.0)		n. a.	P = 0.259	P = 0.259
Not cohabiting	5 (3.2)							
**Gestational age at assessment**								
<18 weeks	34 (21.9)			15 (15.0)			P < 0.001	
18–22 weeks	81 (52.3)		n. a.	84 (84.0)		n. a.		
>22 weeks	40 (25.8)			1 (1.0) ***				
**Time interval from suspicion of fetal anomaly** (**normal scan in comp**. **group**) **to assessment**								
0–2 days	75 (48.4)		n. a.	15 (15.0)		n. a.	P < 0.001	
3–6 days	52 (33.5)			53 (53.0)				
≥7 days	28 (18.1)			32 (32.0)				
Classification of severity**								
1	41 (26.5)							
2	39 (25.2)		n. a.					
3	20 (12.9)							
4	22 (14.2)							
5	33 (21.3)							

* P-values are also presented for the differences for each sex between the two groups (Chi-Square – tests).

** Classification of severity of anomaly.

1: Lethal or serious with no available treatment, with or without prognostic ambiguity (e.g., acrania, skeletal dysplasia with small thorax, holoprosencephaly).

2: Serious with available treatment but all with prognostic ambiguity (e.g. myelomeningocele with hydrocephalus, hypoplastic left heart syndrome). 3: Mild to moderate severity with available treatment, often with good result, but all with prognostic ambiguity (e.g. bilateral clubfoot or cleft lip with no other markers, but condition known to be associated with syndromes not apparent prenatally). 4: Mild to moderate severity with available treatment, often with good result, without prognostic ambiguity (e.g. gastroschisis, unilateral clubfoot). 5: Severity not classified; awaiting clarification, all with prognostic or diagnostic ambiguity. Prognosis highly dependent on the results of an invasive test (e.g. omphalocele, bilateral clubfoot with chromosomal soft markers), or a reliable diagnosis was not available at inclusion due to an incomplete ultrasound examination (e.g. maternal obesity).

*** In the group without fetal anomaly this case has been moved to the 18–22 weeks group due to statistical purposes. This case was included in the study at week 22 + 1 day of gestation.

### Psychometric questionnaires

Participating men completed the questionnaires within a few days after the ultrasound examination at our tertiary referral centre. The questionnaires were completed at the hospital (a few men in the comparison group filled out the questionnaires at home) and the participants were instructed not to discuss their responses before completion. Social dysfunction and health perception (somatic symptoms) were assessed by the corresponding subscales of General Health Questionnaire (GHQ-28) [10]. Endpoints of psychological distress were assessed by the anxiety and depression subscales of GHQ-28, Impact of Events Scale (IES-22) [11], [12], and Edinburgh Postnatal Depression Scale (EPDS) [13].

IES-22 measures three subscales of psychological and behavioural distress during the previous week: intrusion (7 items), avoidance (8 items), and arousal (7 items). Intrusion is characterised by unbidden thoughts and images, troubled dreams, strong waves of feelings, and repetitive behaviour, related to the experience of knowing about the fetal condition. Avoidance includes ideational constriction related to the fetal condition, denial of the consequences of the anomaly, blunted sensations, behavioural inhibition, and emotional numbness. Arousal measures distress-associated psycho-physiological activation and includes items addressing anger and irritability, a heightened startle response, concentration difficulties, and hypervigilance. Each IES item has a score range of 0–5 yielding a scoring range of 0–35 for intrusion and arousal, and 0–40 for avoidance. A score < 9 in any of the dimensions was considered to be within the normal range, 9–19 was defined as a moderate response, while 20 or more indicated intrusion, avoidance, or arousal of clinical importance [14].

GHQ-28 has four subscales, each with seven items, measuring social dysfunction, health perception (somatic symptoms) and psychological distress (anxiety and severe depressive symptoms) during the preceding two weeks. Likert scoring (item scores 0-1-2-3, total range 0–84) was used to compare distress levels within and between groups. Case score is a dichotomous scoring method (item scores 0-0-1-1). The GHQ-28 sum case score provides an estimate of the prevalence of clinically significant psychological distress, defined as a sum case score ≥ 6 (total range 0–28). Clinically significant depression was defined as a GHQ-28 depression subscale case score ≥ 2. GHQ items 24, 25, 27, and 28, were used to assess suicidal ideation [15].

The EPDS consists of ten questions and is a self-rating scale designed to detect postnatal depression. Five items measure dysphoric mood, two measure anxiety, and three (one per item) measure guilt, suicidal ideas, and “not coping” experienced during the previous week. The EPDS-10 has been validated for use during pregnancy, including men [16], [17]. We scored the EPDS-10 with Likert’s score (item score 0-1-2-3; total sum score range 0–30). EPDS sum score ≥ 10 was considered to be associated with mild depression and a score ≥ 13 was used to identify at least moderate depression [18], [19].

### Statistics

Sample size calculation was based on Skari et al. [3] who reported that GHQ sum Likert scores differed by ⅔ SD for parents of fetuses diagnosed at 25 – 30 weeks of gestational age compared to those diagnosed earlier in pregnancy. Each gestational age group (see Table 1) would require 40 patients to obtain the same difference with α = 0.05 and a statistical power of 85%. Effect size was assessed by Cohen’s d.

The questionnaires were optically readable. Completed questionnaires were scanned with Cardiff TeleForm version 10.1 (Autonomy Corporation plc, Cambridge, England), and stored in Access version 97 (Microsoft Corporation, Redmond, Washington). Calculations were performed with SPSS version 18.0 (Statistical Package for the Social Sciences, SPSS Inc., Chicago, Illinois).

For descriptive statistics, we used parametric or non-parametric analyses, as appropriate. Analysis of variance (ANOVA) was used to identify predictors of psychosocial distress, including the categorical (non-ordinal) variable on classification of severity with ambiguity, paternal age, number of previous children, education, gestational age at inclusion, and time interval from suspicion of fetal anomaly to assessment. Continuous variables were transformed into categorical variables (relevant groups). We made cross-tabulations concerning possible interactions between the background variables. The requirements of minimum expected cell frequency were fulfilled. To study correlation between men and women (within the couple) we used Spearman’s Rho.

ANOVA was first performed with each of the independent variables separately. Subsequently, a full analysis was run of all the relevant independent variables with all possible two-way interaction effects for each of the responses. Interaction effects with p > 0.1 were excluded. After reanalysing the data, the interaction effects with p < 0.05 were included in the final model. To control the overall significance of the test for those variables with more than two levels we used Tukey’s HSD post hoc test for detailed analyses of the effects. This methodology is underlying all statements in the results section when more than one level was used. Standard residual plots were used for model evaluation. Levene’s test of equality of error variances was used to test the assumptions underlying the analysis of variance. We used a significance level > 0.05 to avoid violation of the assumption of homogeneity of variance.

### Ethical issues

The study was approved by the Regional Ethics Committee of Southern Norway December 21^st^ 2005 (Reference number S-05281). Written informed consent was obtained before participation. In accordance with the study protocol, any participant with a case score of ‘1’ on items addressing suicidal ideation was contacted for clinical evaluation within the same day, and if necessary, offered psychiatric assistance.

## Results

The mean paternal age was almost the same in the study- and comparison group; 33 (SD 6) and 34 years (SD 5), respectively. Age within gender category did not differ between the groups (p > 0.120). In both groups men were significantly older than women (p < 0.001). In the study group, more men than women had junior college or less (52% versus 35%, p < 0.001). There was no such difference in education in the comparison group. For other background variables see Table 1.

All psychometric responses were higher in the study group than in the comparison group (Table 2). Within both groups all psychometric responses were significantly lower in men than in women, except for GHQ depression and IES avoidance in the comparison group. These two scores also had a small effect size (Table 2). The differences between the groups and genders are also illustrated by the number of men and women who scored above conventional cut-off levels (Table 3).

**Table 2:** **Psychometric scores for men and women within each group**, **i**.**e**. **with and without fetal anomaly**

	**Fetal anomaly (N = 155)**			**No fetal anomaly (N = 100)**			**With and without fetal anomaly (N = 255)**	
							**Men versus men**	**Women versus women**
	**Men**	**Women**	P-value*	**Men**	**Women**	P-value*	P-value**	P-value**
	**Median (min-max)**	**Median (min-max)**	**Cohen’s d**	**Median (min-max)**	**Median (min-max)**	**Cohen’s d**	**Cohen’s d**	**Cohen’s d**
	**Mean (SD)**	**Mean (SD)**		**Mean (SD)**	**Mean (SD)**			
**GHQ sum Likert score**	19 (2–52)	26 (8–61)	P < 0.001	14 (4–41)	19 (8–57)	P < 0.001	P < 0.001	P < 0.001
	21.4 (9.7)	27.6 (11.5)	d = -0.583	15.3 (6.1)	19.8 (8.2)	d = -0.623	d = 0.753	d = 0.780
**GHQ somatisation**	5 (1–18)	7 (0–18)	P < 0.001	4 (1–21)	6 (0–16)	P < 0.001	P = 0.008	P = 0.008
	5.4 (3.3)	7.4 (4.0)	d = -0.545	4.5 (3.4)	6.1 (3.4)	d = -0.471	d = 0.269	d = 0.350
**GHQ social dysfunction**	7 (3–19)	9 (5–17)	P < 0.001	7 (0–17)	7 (3–19)	P < 0.001	P < 0.001	P < 0.001
	8.1 (2.5)	9.5 (2.8)	d = -0.527	6.5 (2.1)	8.1 (2.6)	d = -0.677	d = 0.693	d = 0.581
**GHQ anxiety**	6 (0–17)	8 (0–18)	P < 0.001	3 (0–13)	5 (0–14)	P = 0.001	P < 0.001	P < 0.001
	6.6 (3.7)	8.8 (4.3)	d = -0.548	4.0 (2.3)	5.4 (3.2)	d = -0.502	d = 0.844	d = 0.897
**GHQ depression**	0 (0–15) 1.4 (2.6)	0 (0–16)	P = 0.011	0 (0–4)	0 (0–10)	P = 0.791	P < 0.001	P < 0.001
		1.9 (3.1)	d = -0.175	0.3 (0.7)	0.4 (1.2)	d = -0.102	d = 0.578	d = 0.638
**GHQ sum case score**	3 (0–20) 4.6 (5.0)	7 (0–26)	P < 0.001	0 (0–15)	3 (0–20)	P < 0.001	P < 0.001	P < 0.001
		8.0 (6.3)	d = -0.598	1.7 (2.7)	4.5 (4.2)	d = -0.793	d = 0.722	d = 0.654
**IES intrusion**	17 (0–35)	25 (1–35)	P < 0.001	6 (0–24)	8 (0–27)	P = 0.002	P < 0.001	P < 0.001
	16.5 (8.7)	22.5 (8.3)	d = -0.706	6.7 (5.6)	9.4 (6.3)	d = -0.453	d = 1.340	d = 1.778
**IES avoidance**	7 (0–37) 9.2 (7.8)	11 (0–34)	P = 0.001	0 (1–18)	0.5 (0–26)	P = 0.397	P < 0.001	P < 0.001
		11.3 (7.1)	d = -0.282	1.7 (2.9)	2.2 (3.9)	d = -0.145	d = 1.275	d = 1.589
**IES arousal**	7 (0–31)	14 (0–35)	P < 0.001	2 (0–12)	3 (0–20)	P = 0.019	P < 0.001	P < 0.001
	9.1 (7.1)	14.8 (9.0)	d = -0.703	2.6 (3.0)	3.9 (4.2)	d = -0.356	d = 1.193	d = 1.552
**EPDS sum**	7 (0–24)	12 (0–29)	P < 0.001	1 (0–9)	2 (0–14)	P < 0.001	P < 0.001	P < 0.001
	7.5 (5.2)	12.5 (5.9)	d = -0.899	1.4 (1.9)	3.0 (2.9)	d = -0.653	d = 1.558	d = 2.044

P-values and Cohen’s d are presented for the differences between men and women within each group and for each sex between the two groups.

* Wilcoxon Signed Ranks Test (non parametric, related samples).

** Independent Samples Mann–Whitney U tests (non parametric, unrelated samples).

Abbreviations:

*EPDS *Edinburgh Postnatal Depression Scale.

*GHQ *General Health Questionnaire (28 items).

*IES *Impact of Event Scale (22 items).

**Table 3:** **Number of men and women within each group** (**i**.**e**. **with and without fetal anomaly**) **above clinically accepted psychometric cut**-**off values**

	**Fetal anomaly (N = 155)**			**No fetal anomaly (N = 100)**			**With and without fetal anomaly (N = 255)**	
							**Men versus men**	**Women versus women**
	**Men**	**Women**	P-value*	**Men**	**Women N (%)**	P-value*	P-value*	P-value*
	**N (%)**	**N (%)**		**N (%)**				
**GHQ sum case score **≥**6**	50 (32.3)	88 (56.8)	P = 0.001	8 (8.0)	37 (37.0)	P = 0.240	P < 0.001	P = 0.003
**IES intrusion **≥**20**	65 (41.9)	112 (72.3)	P < 0.001	2 (2.0)	8 (8.0)	P = 0.371	P < 0.001	P < 0.001
**IES avoidance **≥**20**	18 (11.6)	23 (14.8)	P = 0.007	1 (1.0)	1 (1.0)	n.a.	P = 0.001	P < 0.001
**IES arousal **≥**20**	13 (8.4)	44 (28.4)	P < 0.001	1 (1.0)	1 (1.0)	n.a.	P = 0.008	P < 0.001
**EPDS sum **≥**13**	28 (18.1)	71 (45.8)	P = 0.006	0	2 (2.0)	n.a.	P < 0.001	P < 0.001

P-values are presented for the differences between men and women within each group and for each sex between the two groups.

* Chi square.

Abbreviations:

*EPDS *Edinburgh Postnatal Depression Scale.

*GHQ *General Health Questionnaire.

*IES *Impact of Event Scale.

**Figure 1:**
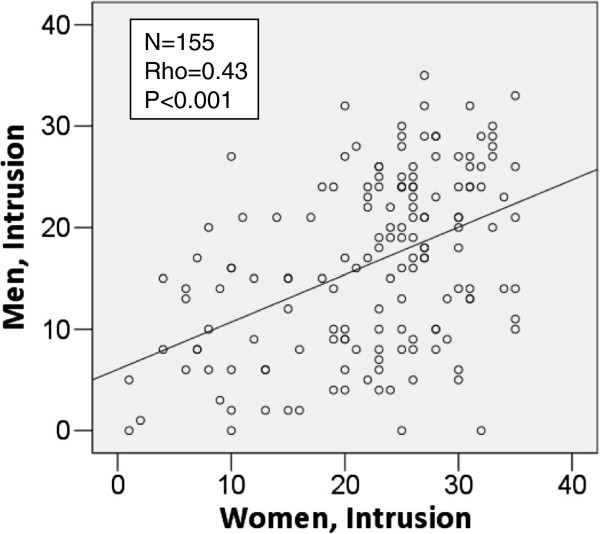
**Correlations for IES Intrusion within couples in the fetal anomaly group; scatter plot including regression line and correlation statistics (Spearman’s Rho). **The plot represents paired samples (within the couple). Abbreviations: IES; Impact of Event Scale.

In the study group the correlations in psychometric scores within the couples were highly significant for all psychometric endpoints (i.e. IES intrusion, avoidance, arousal, GHQ sum Likert, and EPDS sum) with p < 0.001, see one example, Figure 1. No significant correlations were observed in the comparison group.

We performed unadjusted ANOVAs for the men in the study group, with six independent variables (i.e., severity of fetal anomaly, paternal age, number of previous children, education, gestational age at assessments, and time interval from suspicion of fetal anomaly to assessments). The outcome variables were IES intrusion, avoidance, and arousals, GHQ sum Likert, and EPDS sum. Fetal diagnostic and prognostic severity with inherent ambiguity was the only explanatory factor that reached statistical significance concerning three of the psychometric measures (Tables 4 and 5).

**Table 4:** Unadjusted and adjusted values of the subscale scores from the impact of event scale as dependent variable for the men in the fetal anomaly group

**Independent variable**		**Dependent variable**											
		**IES intrusion (N = 155)**				**IES avoidance (N = 155)**				**IES arousal (N = 155)**			
		**Unadjusted**		**Adjusted**		**Unadjusted**		Adjusted*		**Unadjusted**		**Adjusted**	
		**Mean (CI)**	**P-value**	**Mean (CI)**	**P-value**	**Mean (CI)**	**P-value**	**Mean (CI)**	**P-value**	**Mean (CI)**	**P-value**	**Mean (CI)**	**P-value**
Fetal diagnostic and prognostic classification; Category 1–5 (see text, footnotes, Table 1)	1	18.2 (15.7-20.7)	< 0.001	19.1 (16.3-21.9)	< 0.001**	11.2 (8.8-13.6)	0.072	10.3 (7.9-12.7)	0.024 ***	10.2 (8.1-12.3)	0.097	10.4 (8.0-12.8)	0.004†
	2	19.7 (17.1-22.3)		19.8 (16.9-22.6)		9.8 (7.4-12.2)		9.1 (6.7 -11.5)		11.2 (9.0-13.3)		11.3 (8.9-13.7)	
	3	17.1 (13.4-20.7)		16.4 (12.6-20.2)		9.4 (6.0-12.7)		8.8 (5.6-12.0)		9.7 (6.7-12.7)		9.6 (6.4-12.8)	
	4	9.5 (6.1-13.0)		9.5 (5.9-13.2)		5.5 (2.2-8.7)		3.9 (0.7-7.1)		4.4 (1.5-7.2)		4.0 (0.9-7.0)	
	5	15.0 (12.2-17.8)		15.6 (12.6-18.7)		8.2 (5.6-10.9)		7.2 (4.6-9.8)		8.2 (5.8-10.5)		8.2 (5.6-10.7)	
Paternal age (years)	19-30	15.4 (13.1-17.7)	0.514	16.2 (13.6-18.7)	0.887	9.0 (6.9-11.0)	0.461 ††	8.7 (6.5-10.9)	0.548	9.4 (7.5-11.3)	0.647	9.7 (7.5-11.8)	0.471
	31–35	17.1 (14.8-19.5)		15.6 (13.2-18.1)		9.9 (7.8-12.0)		7.9 (5.8-9.9)		9.5 (7.6-11.5)		8.6 (6.6-10.7)	
	36-67	17.0 (14.5-19.6)		16.5 (13.8-19.1)		8.0 (5.7-10.2)		7.0 (4.8-9.2)		8.3 (6.2-10.4)		7.8 (5.5-10.0)	
Previous children	Yes	17.5 (15.5-19.4)	0.159	16.9 (14.8-19.0)	0.257	9.7 (7.9-11.4)	0.410	8.3 (6.5-10.1)	0.463	9.3 (7.7-10.9)	0.741	9.0 (7.2-10.7)	0.635
	No	15.5 (13.4-17.5)		15.3 (13.1-17.4)		8.6 (6.8-10.4)		7.4 (5.6-9.2)		8.9 (7.3-10.6)		8.4 (6.6-10.2)	
Education	≤Junior college	16.9 (15.0-18.8)	0.540	16.2 (14.1-18.2)	0.901	9.6 (7.9-11.3)	0.450	8.2 (6.5-9.9)	0.557	9.1 (7.5-10.6)	0.934	8.3 (6.6-10.0)	0.496
	>Junior college	16.0 (14.0-18.1)		16.0 (13.9-18.1)		8.7 (6.8-10.5)		7.5 (5.7-9.3)		9.2 (7.5-10.8)		9.1 (7.3-10.9)	
Gestational age at inclusion (weeks)	<18	14.9 (12.0-17.9)	0.469	14.7 (11.5-17.8)	0.323	8.1 (5.5-10.8)	0.633	6.8 (4.2-9.5)	0.413	8.5 (6.1-10.9)	0.821	8.3 (5.7-10.9)	0.897
	18–22	16.9 (14.9-18.8)		15.8 (13.6-17.9)		9.7 (7.9-11.4)		8.8 (7.0-10.6)		9.4 (7.8-10.9)		8.6 (6.8-10.4)	
	>22	17.2 (14.5-19.9)		17.9 (15.1-20.6)		9.0 (6.6-11.4)		7.9 (5.6-10.3)		9.1 (6.9-11.3)		9.1 (6.9-11.4)	
Time interval from suspicion of fetal anomaly to assessment (days)	0-2	15.6 (13.6-17.6)	0.277	15.2 (13.1-17.2)	0.599	9.1 (7.4-10.9)	0.997	8.4 (6.6-10.1)	0.741	9.2 (7.6-10.9)	0.770	8.9 (7.1-10.6)	0.765
	3–6	16.8 (14.4-19.1)		15.9 (13.5-18.4)		9.1 (7.0-11.3)		7.3 (5.2-9.5)		8.6 (6.6-10.5)		8.1 (6.0-10.1)	
	≥7	18.6 (15.4-21.9)		17.2 (13.9-20.5)		9.3 (6.3-12.2)		7.9 (5.0-10.7)		9.8 (7.1-12.4)		9.1 (6.3-11.9)	

Abbreviations:

*CI *Confidence interval 95%, *IES *Impact of Event Scale.

The analyses are performed using ANOVA (Analysis of variance) in the group of men in the study group (with fetal anomaly).

* Left out three outliers due to violation to the significant level of Levene’s test.

** Tukey Honestly Significant Difference (Tukey HSD) test: significant difference between category 4 and 1, p = 0.001; 4 and 2, P < 0.001; 4 and 3, P = 0.032.

*** Tukey HSD test: significant difference between category 4 and 1, P = 0.009; 4 and 2, P = 0.037.

† Tukey HSD test: significant difference between category 4 and 1, P = 0.016; 4 and 2, P = 0.003.

†† Two outliers removed to not violate the Levene’s test of equality of error variance.

**Table 5:** Unadjusted and adjusted values of general health questionnaire sum Likert score and Edinburgh postnatal depression scale sum score as dependent variables for the men in the fetal anomaly group

**Independent variable**		**Dependent variable**							
		**GHQ sum Likert score (N = 155)**				**EPDS sum score (N = 155)**			
		**Unadjusted**		Adjusted*		**Unadjusted**		Adjusted**	
		**Mean (CI)**	**P-value**	**Mean (CI)**	**P-value**	**Mean (CI)**	**P-value**	**Mean (CI)**	**P-value**
Fetal diagnostic and prognostic classification; category 1–5 (see text, footnotes, Table 1)	1	22.9 (19.8-25.9)	0.015	24.8 (21.4-28.2)	0.426	9.0 (7.5-10.5)	0.001	9.6 (7.7-11.5)	0.001***
	2	21.7 (18.6-24.8)		20.9 (17.6-24.3)		8.4 (6.9-10.0)		6.6 (4.6-8.7)	
	3	21.6 (17.2-25.9)		21.1 (16.7-25.6)		8.6 (6.4-10.8)		6.5 (3.8-9.2)	
	4	19.2 (15.1-23.4)		21.0 (16.5-25.5)		4.1 (2.0-6.2)		3.0 (0.7-5.4)	
	5	20.6 (17.2-24.0)		21.7 (18.2-25.2)		5.9 (4.2-7.6)		5.8 (3.8-7.8)	
Paternal age (years)	19-30	21.9 (19.3-24.5)	0.230	23.6 (19.9-27.2)	0.302	7.8 (6.4-9.2)	0.561	6.9 (5.3-8.5)	0.599
	31–35	22.8 (20.1-25.4)		22.3 (19.5-25.1)		7.7 (6.2-9.1)		6.3 (4.8-7.9)	
	36-67	19.5 (16.7-22.3)		19.9 (16.7-23.1)		6.8 (5.2-8.3)		5.7 (3.9-7.6)	
Previous children	Yes	21.5 (19.3-23.6)	0.964	22.1 (19.6-24.5)	0.872	7.4 (6.2-8.6)	0.887	6.5 (5.1-7.8)	0.739
	No	21.5 (19.3-23.8)		21.8 (19.2-24.3)		7.5 (6.3-8.7)		6.2 (4.7-7.6)	
Education	≤Junior college	22.0 (19.8-24.1)	0.516	22.7 (20.3-25.1)	0.353	7.7 (6.6-8.9)	0.487	6.1 (4.7-7.4)	0.568
	>Junior college	20.9 (18.6-23.2)		21.1 (18.6-23.7)		7.1 (5.9-8.4)		6.6 (5.2-8.0)	
Gestational age at inclusion (weeks)	<18	19.8 (16.5-23.1)	0.556	20.5 (16.9-24.2)	0.502	5.9 (4.1-7.6)	0.136	4.8 (2.6-6.9)	0.123
	18–22	21.8 (19.7-24.0)		22.9 (20.4-25.5)		8.0 (6.8-9.1)		6.6 (5.3-7.9)	
	>22	21.9 (18.8-24.9)		22.2 (19.1-25.4)		7.8 (6.1-9.4)		7.6 (5.9-9.3)	
Time interval from suspicion of fetal anomaly to assessment (days)	0-2	20.6 (18.4-22.8)	0.143	20.3 (17.9-22.7)	0.157	7.4 (6.2-8.6)	0.784	6.4 (5.0-7.7)	0.880
	3–6	20.8 (18.2-23.5)		20.6 (17.7-23.5)		7.2 (5.8-8.6)		6.0 (4.4-7.6)	
	≥7	24.7 (21.1-28.3)		24.8 (20.8-28.9)		8.0 (6.1-10.0)		(4.5-8.6)	

Abbreviations:

*CI *Confidence interval 95%, *EPDS *Edinburgh Postnatal Depression Scale, *GHQ *General Health Questionnaire.

The analyses are performed using ANOVA (Analysis of variance) in the group of men in the study group (with fetal anomaly).

* Interaction: Paternal age* Time interval from suspicion of fetal anomaly to inclusion, P = 0.016.

** Interaction: Fetal diagnostic and prognostic classifications* Gestational age at inclusion, P = 0.017; Paternal age* Previous children, P = 0.023.

*** Tukey Honestly Significant Difference (Tukey HSD) test: test: significant difference between category 4 and 1, P = 0.001; 4 and 2, P = 0.007; 4 and 3, P = 0.021.

Adjusted ANOVAs in the study group of men with the same explanatory variables showed that diagnostic and prognostic severity of fetal anomaly was significant for all IES subscales (i.e. intrusion, p < 0.001; avoidance, p = 0.024; arousal, p = 0.004) and sum EPDS (p = 0.001). GHQ sum Likert did not reach significance (p = 0.426). There were no consistent patterns in the interactions among components, see Table 5.

Post hoc tests (Tukey HSD test) showed that the men in the category “Mild to moderate severity with available treatment, often with good result, without prognostic ambiguity” had significantly lower scores than the men in the other categories as presented in Table 4 for IES and Table 5 for EPDS.

## Discussion

Expectant fathers experienced psychological distress after prenatal detection of a fetal anomaly. We observed a significant difference in all psychometric assessments between the men in the fetal anomaly group and the men in the group with no fetal anomaly, e.g. mean (SD) values for IES intrusion were 16.5 (8.7) and 6.7 (5.6), respectively. Distress levels were lower than for the women in the respective groups. This pattern concurs with findings by Skari et al. [15] in fathers after the birth of a healthy baby versus a baby with a prenatally detected congenital malformation [3]. Epidemiological studies of distress have also reported lower scores in men than in women [20]. The reason for this dissimilarity may be due to gender-specific differences in the experience of pregnancy and the biology of reproduction; paternal experience has to either be through the woman or to be theoretical [21].

In our study the severity of the fetal malformation and ambiguity concerning diagnosis and prognosis were the only predictors of paternal psychological distress, social dysfunction and health perception. Neither gestational age nor any of the other background variables influenced the paternal psychological reaction, as they do in pregnant women [1].

After prenatal detection of a fetal anomaly both parents will experience psychological shock and acute grief [22]. In our study, the only independent variable that predicted paternal stress levels was the prognostic classification of fetal anomalies, as shown in Tables 4 and 5. For many of the cases, the prognosis was ambiguous because the prenatal diagnosis was known to have significant inherent prognostic variation. At the time of psychometric assessment 33 pregnant women and their partners were awaiting further diagnostic classification, either because the diagnosis was highly dependent on the results of an invasive test or because the ultrasonographic examination was technically incomplete. The counseling was performed on the basis of these uncertainties. The category with best prognosis and no ambiguity (category 4) caused the lowest distress levels, as expected. In the adjusted analyses the p-values were < 0.025 for all distress scores with the exception of GHQ sum Likert score (Tables 4 and 5). GHQ measures general health perception, social dysfunction and psychological distress during the last two weeks, and is not related to any specific event. This may explain why this measure (GHQ sum Likert score) did not reach significance, even though the score in the fetal anomaly group was elevated compared to the group without fetal anomaly.

Matthey et al. [16] discussed the antenatal use of EPDS to measure mood in expectant fathers. They concluded that EPDS is a reliable and valid measure, but that the cut-off point for clinical significant psychological distress level in fathers is two points lower than in mothers for depression and anxiety. Their argument is the question on crying, in which there was a significant difference in endorsement between men and women, “- men tend not to cry when unhappy, but rather express their unhappiness through other behaviors” [16]. In our study there was a considerable gender difference; 15% of the men compared to 60% of the women in the study group had a score of two (yes, very often) or three (yes, almost all the time) for the EPDS question: “Have you in the last seven days been so unhappy that you have been crying?” In the comparison group none of the men and only one woman scored in this range.

The lower scores in men compared to women for IES and GHQ might be due to the same mechanism, i.e. gender differences in the response to some of the questions. However in an evaluation of IES after 20 years of use, no gender difference was detected [23]. Goulia et al. assessed distress in younger and older patients with chronic medical conditions using GHQ-28 [24]. Stress levels were not influenced by gender [24]. Thus, the demonstrated gender difference in distress levels in our study might be pregnancy-specific.

In contrast, Clarke et al. argue that there is evidence in social psychology research literature for females generally reporting higher levels of depression, anxiety and psychosomatic stress than males [25]. These differences are the result of several factors including cultural and societal expectations, biological mechanisms, and care giving practices. From our study we are not able to state why men have lower scores than women. Men and women respond differently to stressful events, including those relating to parenting and chronic illness in a child [25]. A retrospective study found that mothers reported higher levels of stress than fathers only during the early stages of children’s illness and active treatment, and argued that gender differences diminish over time [26].

A study from Australia and New Zealand describes how first time fathers experience significant distress even in normal pregnancies [5]. It highlights vulnerability in the transition to fatherhood if the relationship with the partner and the social network is poor. Pregnancy-related paternal depression or anxiety is a new research agenda and there is no consensus on this issue [6]. In our study we did not investigate social network or relationship with the partner during pregnancy.

The background variables age, educational level and number of previous children were significantly different between men and women in the two groups, but only education was different between the men in the two groups. The men tended to be older than the women and the women had higher educational level than the men. These trends are consistent with the traditional societal pattern in Norway over the last decades. It is unlikely that these differences can explain the difference in psychological stress scores between men and women or between the men in the two groups.

The correlation of psychometric measurements within couples in the study group (Figure 1) is probably explained by the severity of fetal anomaly; this is the same for each couple and is the independent variable with the greatest explanatory value to the psychological distress in both men and women [1]. Additionally, there was a lack of correlations between men and women in the comparison group. The comparison group only scored on a limited interval or range of the scales for distress measurements. This might be the reason why it is not possible to detect a significant correlation between men and women in a sample of 100 couples in normal pregnancies.

A weakness of the study is that we do not have distress measurements prior to the event, i.e. prior to the ultrasound examination detecting fetal anomaly. Due to the unpredictable occurrence of fetal anomalies this was not feasible. The strength of the study is the sample size, the incorporation of a comparison group, and the use of validated and widely applied questionnaires.

The selection of instruments to assess psychological response during pregnancy may be subject to discussion [27], although without any clear recommendations so far. We have chosen a broad variety of instruments covering general health perception, social dysfunction, key components of psychological distress, the response to a specific stressful event related to detection of fetal anomaly, and one questionnaire validated for use during pregnancy in both men and women. The chosen questionnaires have been used in other related studies, thereby enabling the comparison of results.

## Conclusion

Despite the frequent use of ultrasound examination in pregnancy, little attention has been devoted the psychological response of the expectant father to the detection of fetal anomalies. Men in the group with fetal anomaly had significantly higher scores on all distress measures than the men in the group without fetal anomaly. The severity of anomaly and diagnostic and prognostic ambiguity influenced the paternal psychological response. Although men had lower scores than women on all psychometric outcomes, the correlation of distress levels associated with the detection of fetal anomalies within couples was high. This knowledge may facilitate intensified support for both expectant parents in situations of detected fetal anomaly and thus help reduce strain within the family.

## Abbreviations

EPDS: Edinburgh postnatal depression scale; GHQ: General health questionnaire; IES: Impact of event scale.

## Competing interests

The authors declare that they have no competing interests.

## Authors’ contributions

AK planned and performed the study, analysed the data, and wrote the paper. AH participated in performing the study and writing of the paper. UFM participated in planning the study and writing of the paper. TN participated in analyzing the data and writing of the paper. HS participated in planning the study and writing of the paper. GH participated in planning and performing the study, and writing of the paper. All authors read and approved the final manuscript.

## Pre-publication history

The pre-publication history for this paper can be accessed here:

http://www.biomedcentral.com/1471-2393/13/147/prepub

## References

[B1] Kaasen A Helbig A Malt UF Naes T Skari H Haugen G Acute maternal social dysfunction, health perception and psychological distress after ultrasonographic detection of a fetal structural anomaly BJOG 2010 117 1127 1138 10.1111/j.1471-0528.2010.02622.x 20528866

[B2] Tifferet S Manor O Constantini S Friedman O Elizur Y Sex difference in paternal reaction to pediatric illness J Child Health Care 2011 15 118 125 10.1177/1367493510397710 21685227

[B3] Skari H Malt UF Bjornland K Egeland T Haugen G Skreden M Prenatal diagnosis of congenital malformations and parental psychological distress–a prospective longitudinal cohort study Prenat Diagn 2006 Nov 26 1001 1009 10.1002/pd.1542 16958144

[B4] Ekelin M Crang SE Larsson AK Nyberg P Marsal K Dykes AK Parental expectations, experiences and reactions, sense of coherence and grade of anxiety related to routine ultrasound examination with normal findings during pregnancy Prenat Diagn 2009 29 952 959 10.1002/pd.2324 19582763

[B5] Condon JT Boyce P Corkindale CJ The first-time fathers study: a prospective study of the mental health and wellbeing of men during the transition to parenthood Aust N Z J Psychiatry 2004 38 56 64 10.1111/j.1440-1614.2004.01298.x 14731195

[B6] Schumacher M Zubaran C White G Bringing birth-related paternal depression to the fore Women Birth 2008 21 65 70 10.1016/j.wombi.2008.03.008 18479990

[B7] Boyce P Condon J Barton J Corkindale C First-time fathers' study: psychological distress in expectant fathers during pregnancy Aust N Z J Psychiatry 2007 41 718 725 10.1080/00048670701517959 17687657

[B8] Hunfeld JA Tempels A Passchier J Hazebroek FW Tibboel D Brief report: parental burden and grief one year after the birth of a child with a congenital anomaly J Pediatr Psychol 1999 24 515 520 10.1093/jpepsy/24.6.515 10608103

[B9] Fleiss JL Measuring nominal scale agreement among many raters Psychol Bull 1971 76 378 382

[B10] Goldberg DP Hillier VF A scaled version of the general health questionnaire Psychol Med 1979 9 139 145 10.1017/S0033291700021644 424481

[B11] Horowitz M Wilner N Alvarez W Impact of event scale: a measure of subjective stress Psychosom Med 1979 41 209 218 472086 10.1097/00006842-197905000-00004

[B12] Weiss D Marmar C Wilson J, Keane T The Impact of Event Scale -revised Assessing psychological trauma and PTSD 1997 New York: Guildford

[B13] Cox JL Holden JM Sagovsky R Detection of postnatal depression. Development of the 10-item Edinburgh postnatal depression scale Br J Psychiatry 1987 150 782 786 10.1192/bjp.150.6.782 3651732

[B14] Tjemsland L Soreide JA Malt UF Posttraumatic distress symptoms in operable breast cancer III: status one year after surgery Breast Cancer Res Treat 1998 47 141 151 10.1023/A:1005957302990 9497102

[B15] Skari H Skreden M Malt UF Dalholt M Ostensen AB Egeland T Comparative levels of psychological distress, stress symptoms, depression and anxiety after childbirth–a prospective population-based study of mothers and fathers BJOG 2002 109 1154 1163 12387470 10.1111/j.1471-0528.2002.00468.x

[B16] Matthey S Barnett B Kavanagh DJ Howie P Validation of the Edinburgh postnatal depression scale for men, and comparison of item endorsement with their partners J Affect Disord 2001 64 175 184 10.1016/S0165-0327(00)00236-6 11313084

[B17] Murray D Cox JL Screening for depression during pregnancy with the Edinburgh depression scale (EPDS) J Reprod Infant Psychol 1990 8 99 107 10.1080/02646839008403615

[B18] Boyce PM Stubbs J Todd AL The Edinburgh postnatal depression scale: validation for an Australian sample Aust N Z J Psychiatry 1993 27 472 476 10.3109/00048679309075805 8250792

[B19] Murray L Carothers AD The validation of the Edinburgh post-natal depression scale on a community sample Br J Psychiatry 1990 157 288 290 10.1192/bjp.157.2.288 2224383

[B20] Donath S The validity of the 12-item general health questionnaire in Australia: a comparison between three scoring methods Aust N Z J Psychiatry 2001 35 231 235 10.1046/j.1440-1614.2001.00869.x 11284906

[B21] Condon J What about dad? Psychosocial and mental health issues for new fathers Aust Fam Physician 2006 35 690 692 16969437

[B22] Statham H Solomou W Chitty L Prenatal diagnosis of fetal abnormality: psychological effects on women in low-risk pregnancies Baillieres Best Pract Res Clin Obstet Gynaecol 2000 14 731 747 10985942 10.1053/beog.2000.0108

[B23] Sundin EC Horowitz MJ Horowitz's impact of event scale evaluation of 20 years of use Psychosom Med 2003 65 870 876 10.1097/01.PSY.0000084835.46074.F0 14508034

[B24] Goulia P Papadimitriou I Machado MO Mantas C Pappa C Tsianos E Does psychological distress vary between younger and older adults in health and disease? J Psychosom Res 2012 72 120 128 10.1016/j.jpsychores.2011.11.011 22281453

[B25] Clarke NE McCarthy MC Downie P Ashley DM Anderson VA Gender differences in the psychosocial experience of parents of children with cancer: a review of the literature Psycho-oncol 2009 18 907 915 10.1002/pon.1515 19319828

[B26] Freeman K O'Dell C Meola C Childhood brain tumors: parental concerns and stressors by phase of illness J Pediatr Oncol Nurs 2004 21 87 97 10.1177/1043454203262691 15125552

[B27] Meades R Ayers S Anxiety measures validated in perinatal populations: a systematic review J Affect Disord 2011 133 1 15 10.1016/j.jad.2010.10.009 21078523

